# The Effect of Sickle Cell Hemoglobinopathy on Pregnancy, Labor, Puerperium, and Fetal Outcome: A Retrospective Cohort Study From a Single Centre

**DOI:** 10.7759/cureus.34318

**Published:** 2023-01-28

**Authors:** Surbhi Rajauria, Charu Batra Atreja, Anshu Mujalda, Jagdish Mujalda, Shikha Yadav, Ramesh K Kundal

**Affiliations:** 1 Pathology, Maharishi Markandeshwar Institute of Medical Sciences and Research (MMIMSR), Ambala, IND; 2 Obstetrics and Gynaecology, Maharishi Markandeshwar Institute of Medical Sciences and Research (MMIMSR), Ambala, IND; 3 Paediatrics, Military Hospital Ambala, Ambala, IND; 4 Internal Medicine, Military Hospital Ambala, Ambala, IND

**Keywords:** anemia, antenatal screening, fetal outcome, pregnancy, sickle cell disease

## Abstract

Introduction: Sickle cell disease (SCD) is a major risk factor as far as pregnancy and obstetric complications are concerned. It possesses major perinatal and postnatal mortality. The management of pregnancy along with SCD requires a multispecialty team consisting of hematologists, obstetricians, anesthesiologists, neonatologists and intensivists.

Objectives: The objective of this study was to investigate the effect of sickle cell hemoglobinopathy on pregnancy, labor, puerperium, and fetal outcome in the rural and urban localities of Maharashtra, India.

Material and methods: The present study is a comparative retrospective analysis of 225 pregnant women with SCD (genotype AS and SS) and 100 age- and gravida-matched pregnant women with normal hemoglobin (genotype AA) as a control who were treated between June 2013 to June 2015, in Indira Gandhi Government Medical College (IGGMC), Nagpur, India. We analyzed various data regarding obstetrical outcomes and complications in sickle cell disease mothers.

Results: Out of 225 pregnant women, 38 (16.89%) were diagnosed with homozygous sickle cell disease (SS group) while 187 (83.11%) were diagnosed with sickle cell trait (AS group). The most common antenatal complications were sickle cell crisis (17; 44.74%) and jaundice (15; 39.47%) in the SS group whereas pregnancy-induced hypertension (PIH) was noted in 33 (17.65%) in the AS group. Intrauterine growth restriction (IUGR) was recorded in 57.89% of the SS group and 21.39% of the AS group. A higher chance of emergency lower segment cesarean section (LSCS; 66.67% in the SS group and 79.09% in the AS group) was recorded as compared with the control group at 32%.

Conclusion: In order to minimize risks to the mother and fetus and for better outcomes it is prudent to manage pregnancy with SCD vigilantly in the antenatal period. In the antenatal period mothers with this disease should be screened for hydrops or bleeding manifestations such as intracerebral hemorrhage in the fetus. Better feto-maternal outcomes can be achieved by effective multispecialty intervention.

## Introduction

Sickle cell disease (SCD) is a common monogenetic hematological disorder worldwide, affecting an estimated 30 million individuals and representing a major health problem associated with significant morbidity and mortality [1]. In India, SCD accounts for 14.5% of the total number of newborns [2].

SCD occurs in individuals homozygous for the βS globin gene (SS) or heterozygous for the βS allele and different abnormal β globin gene alleles, such as βC (SC), Sβ0 thalassemia, or Sβ+ thalassemia [3]. Pregnant women with SCD are at greater risk of obstetrical complications and perinatal mortality as well as sickle-related complications [4-8]. Complications in the mother as well the fetus include anemia, prepartum and postpartum painful crises, preeclampsia, eclampsia, pulmonary complications, preterm delivery along with other associated risks, and intrauterine growth restriction (IUGR) [9-11].

Many studies have shown that SCD is negatively associated with maternal health and perinatal outcome [12-14]. There are not many studies in India that have explored the risk of poor pregnancy outcomes with SCD [11,15,16]. SCD is prevalent in the tribal populations of Gujarat, Madhya Pradesh, Odisha, Chhattisgarh, Maharashtra, and Rajasthan [16-18]. 

In Maharashtra, the sickle gene is mostly found in all the eastern districts, such as the Vidarbha region, and in some parts of Marathawada. The prevalence of sickle cell carriers in different tribes varies from 0 to 35%.

Kate and Lingojwar [19] recorded a high prevalence of HbS (20-35%) in tribes such as the Bhils, Madias, Pawaras, Pardhans and Otkars of Maharashtra state in India. It has also been estimated that Gadchiroli, Chandrapur, Nagpur, Bhandara, Yavatmal and Nandurbar districts have more than 5000 cases of sickle cell anemia.

The study aimed to compare the outcomes among SCD and sickle cell trait on pregnancy, labor, puerperium, and fetal outcome and non-SCD pregnancy as control.

## Materials and methods

In a comparative retrospective study, a total of 225 pregnant women aged 19 to 40 years attending sickle OPD at Indira Gandhi Government Medical College (IGGMC), Nagpur, India, between June 2013 to June 2015 were observed throughout pregnancy and delivery after acquiring consent from them. Their medical records were analyzed and compared with the result from 100 control pregnant women matched for age and gravida and of similar socioeconomic origin.

A total of 225 SCD cases were studied compared with 100 pregnant age- and gravida-matched control with normal hemoglobin (genotype AA). Amongst the cases were sickle cell trait (AS; 187), and sickle cell anemia (SS; 38).

During their visit to the prenatal clinic, women were included in the study based on their positive SCD status. A thorough history was taken with special emphasis on the obstetric history and significant past history of symptomatology of underlying disease. Abortions were excluded from the study.

Measurement and classification of the variables

Preterm labor was defined as onset of labor before 37 completed weeks of gestation. Anemia was defined as hemoglobin concentration less than 10 gram%. Pregnancy-induced hypertension (PIH) was defined as blood pressure more than 140/90 mmHg on two occasions six hours apart with edema or proteinuria or both. Early neonatal death was defined as neonatal death within seven days of birth. Low birth weight (LBW) was defined as birth weight less than 2500 grams. IUGR was diagnosed when the birth weight was less than the 10th percentile of the average for gestational age. Delayed conception (primary infertility) was diagnosed if a couple failed to achieve pregnancy after one year of unprotected intercourse. Regarding onset of menarche, the first menstrual period usually occurs between the ages of 10-16 years, the average being 13.5 years in India, and an onset after 16 years is considered delayed menarche. The complete proforma followed to study these cases has been given in the Appendix.

Statistical analysis

Cross tabulation method was used to estimate pregnancy outcomes, diagnosis morbidities and treatment received by SCD groups. We excluded the missing values from analysis. To evaluate the risk of these pregnancy outcomes logistic regression was performed. Outcome of each pregnancy and associated conditions were taken as a dependent variable and SCD status as an independent variable.

Pregnant women with normal hemoglobin were compared to pregnant women with SCD and sickle cell traits. Odds ratios were calculated comparing sickle cell trait with SCD. We used SPSS version 19 (IBM Corp., Armonk, NY, USA) for statistical analysis.

## Results

The distribution of pregnant women according to electrophoretic pattern is shown in Table 1. Amongst the total 225 cases, 38 cases were of sickle cell anemia and 187 cases were of sickle cell trait. These cases were compared to 100 individuals in the control group (AA). In the AS group the youngest was 19 years and the oldest was 40 and in the SS group the youngest was 19 and the oldest was 30. Most of the cases in the three groups were in the age range of 21-30 years, i.e. they were adult females of reproductive age. There was no case in the SS group above 30 years, indicating that patients in the SS group were mostly of younger age probably because of their shorter life span as has been observed in the literature (Table 1).

**Table 1:** Showing distribution according to electrophoretic pattern in patients studied

Group	Total cases	Percentage
Sickle cell trait (AS)	187	83.11
Sickle cell anemia / diseases (SS)	38	16.89
Total	225	100

Amongst 225 SCD patients, 104 were primigravidas, 80 were second gravidas, 27 were third gravidas and 14 were fourth gravida or above. In AS patients 44.39% were primigravidas, 39.04% were second gravidas, 10.16% were third gravidas and 6.42% were fourth gravidas or above. In SS patients 55.26% were primigravidas, 18.42% were second gravidas, 21.05% were third gravidas and 5.26% were fourth gravidas or above. They were compared with age- and gravida-matched control with normal hemoglobin. Thus, almost half of the patients were primigravidas in the study group (Table 2).

**Table 2:** Showing gravida-wise distribution of cases. AA: Control group with normal Hb, AS: Sickle cell trait, SS: Sickle cell anemia/diseases, G1: Primary gravida (first pregnancy), G2: Secondary gravida (second pregnancy), G3: Tertiary gravida (third pregnancy), G4: fourth pregnancy

GROUP	G1		G2		G3		G4/more	
	No.	%	No.	%	No.	%	No.	%
AA (n=100)	46	46	40	40	6	6	8	8
AS (n=187)	83	44.39	73	39.04	19	10.16	12	6.42
SS (n=38)	21	55.26	7	18.42	8	21.05	2	5.26

In the present study the mean age of menarche was 15.5 years in the SS group, 13.5 years in the AS group and 12.9 years in the control group. Delayed conception (primary infertility) was found in 10.53% and delayed menarche in 15.79% of the SS group.

The proportion of women who had suffered previous spontaneous abortion was six (6%) in the AA group, 33 (17.64%) in the AS group and four (10.53%) in the SS group. Of the parous patients, the number who had suffered a previous stillbirth was six (6%) in the AA group, 29 (15.51%) in the AS group and nine (23.68%) in the SS group.

Antenatal complications

Anemia was the most common antenatal complication observed in SCD patients and the control group. 94.74% of the SS group patients had moderately severe normocytic normochromic anemia. These patients complained of fatigue and generalized weakness. Blood transfusions were given at some stage to 4% of the control group, 8.02% of the AS patients and 52.63% of the SS patients (Table 3).

**Table 3:** Showing degree of anemia and blood transfusion in study groups AA: Control group with normal Hb, AS: Sickle cell trait, SS: Sickle cell anemia/diseases, BT: Blood transfusion, ()* patients given blood transfusion in each group

Group	Severe anemia		Moderate anemia		Mild anemia		B.T.	
	No.	%	No.	%	No.	%	No.	%
AA (n=100)	18 (4)*	18	63	63	19	19	4	4
AS (n=187)	55 (15)*	29.41	94	50.27	38	20.32	15	8.02
SS (n=38)	23 (18)*	60.53	13 (2)*	34.21	2	5.26	20	52.63

Hemoglobin was estimated in all 225 patients. The hematological profile was studied in 69 AS patients and 23 SS patients compared to 100 age- and gravida-matched controls.

In the SS group the mean values were mean corpuscular volume (MCV) 90.65, mean corpuscular hemoglobin concentration (MCHC) 30.67, red blood cell (RBC) count 2.44, and reticulocyte count (RC) 3.26. The AS and AA groups had comparable hematological profiles (Table 4).

**Table 4:** Hematological profile of AS, SS and control group in Mean SD AA: Control group with normal Hb, AS: Sickle cell trait, SS: Sickle cell anemia/diseases, HCT: hematocrit, MCV: mean corpuscular volume, MCH: mean corpuscular hemoglobin, MCHC: mean corpuscular hemoglobin concentration, RBC: red blood cell, TLC: Total leucocyte count, PC: Platelet count, RC: Reticulocyte count

Haematological indices	AS (n=69)	SS (n=23)	AA (n=100)
Hb (gm %)	8.79+1.61	7.28±1.71	8.66+1.63
HCT (%)	26.25+5.67	22.75±5.51	26.18+4.93
MCV ( f/l)	81.45±8.62	90.65±10.77	78.02+10.54
MCH ( pg)	25.74±3.27	28.99±4.15	25.83+3.49
MCHC ( g/l)	31.09±1.79	30.67±3.04	33.12+2.54
RBC million/ml	3.11±0.83	2.44±0.58	3.35+0.47
TLC cumm	8237.68±2229.62	10952.17±3460.80	8836+1440.5
P.C. lac/cumm	2.11±0.97	2.11±0.72	2.53+0.63
R.C. %	2.46±1.95	3.26±2.84	1.20+0.68

High performance liquid chromatography (HPLC) was done in 20 controls only to find out the average and standard deviation values of F, A, A-2 (Table 5).

**Table 5:** HPLC for quantization of abnormal hemoglobin AA: Control group with normal Hb, AS: Sickle cell trait, SS: Sickle cell anemia/diseases, HPLC: HPLC-high performance liquid chromatography Types of abnormal hemoglobin- S: Hb (S), F: Hb (F), A: Hb (A), A2: Hb (A2)

GROUP	%S	%F	%A	%A2
AA (n=20)	0	0.74+0.58	84.91+4.03	2.27+0.69
AS (n=42)	31.92±3.80	1.24±1.05	56.31±2.94	2.45±0.73
SS (n=15)	69.79±6.69	21.89±4.81	6.67±9.27	1.79±0.9

The single main factor causing significant maternal morbidity with sickle cell anemia was the occurrence of sickle cell crisis in 44.74% of SS patients presenting mostly as acute joint pain and breathlessness (Table 6). Pneumonia was seen in two patients of the SS group. PIH was a common antenatal complication in both groups (17.65% in AS and 18.42% in SS). Mild PIH was more common in the AA group (11%), however severe PIH and preeclampsia was seen in 11% of the AS group and 16% of the SS group. Two patients from the AS group had eclampsia. Urinary tract infection (UTI) was seen in 8% of the AA group, 10.16% of the AS group and 23.68% of the SS group (Table 6).

**Table 6:** Showing antenatal complications in the study group AA: Control group with normal Hb, AS: Sickle cell trait, SS: Sickle cell anemia/diseases, SCC: Sickle cell crisis, PIH: Pregnancy induced hypertension, UTI: Urinary tract infection, TB: tuberculosis

Nature of Complication	AS (n=187)	SS (n=38)	AA (n=100)
Jaundice	6(3.21%)	15(39.47%)	-
SCC	3(1.60%)	17(44.74%)	-
PIH	33(17.65%)	7(18.42%)	11(11%)
UTI	19(10.16%)	9(23.68%)	8(8%)
Pneumonia	-	2(5.26%)	-
Laryngeal T.B.	-	1(2.63%)	-
Piles And Fistula	1(.53%)	-	-
Retroplacental Hematoma	4(2.14%)	-	-
Small Joint Dactylitis	-	2(5.26%)	-

Preterm labor was seen in 8.02% of the AS group and 21.05% of the SS group. Vaginal delivery (VD) was seen in 54% of the AA group compared to 41.17% in the AS group and 60.52% in the SS group. At term, the most common indication in the AS and SS groups for induction of labor was impaired fetal well-being and PIH. Forceps application was either for fetal distress or to curtail prolonged second stage of labor (Table 7).

**Table 7:** Showing mode of delivery AA: Control group with normal Hb, AS: Sickle cell trait, SS: Sickle cell anemia/diseases, VD: Vaginal delivery, LSCS: Lower segment cesarean section

Group	VD		LSCS	
	No.	%	No.	%
AA (n=100)	54	54	46	46
AS (n=187)	77	41.17	110	58.82
SS (n=38)	23	60.52	15	39.47
P value		<0.001		<0.001

Emergency cesarean section (CS) rate was considerably higher in all groups. Fetal distress was the commonest indication in all groups for emergency CS, i.e., 69.57% in the AA group, 79.09% in the AS group and 66.67% in the SS group. Other indications of emergency CS were prolonged labour, premature rupture of membranes (PROM) and PIH. Elective CS in all groups were mostly for IUGR and previous CS (Table 8). CS was considerably higher in all groups, though full-term normal delivery (FTND) was more common in the control group.

**Table 8:** Showing type of cesarean section (CS) in each study group AA: Control group with normal Hb, AS: Sickle cell trait, SS: Sickle cell anemia/diseases * Showing total number of CS in each group

	AA group (n=46)*		AS group (n=110)*		SS group (n=15)*		P value
	No.	%	No.	%	No.	%	
Elective cesarean section	14	30.43	23	20.91	5	33.33	0.321
Emergency cesarean section	32	69.57	87	79.09	10	66.67	0.243

The mean birth weight was 2425 gm in the AA group and 2346.79 gm in the AS group, while in SS patients it was 2036.84 gm. There were two term twin deliveries in the AS group delivered vaginally. In the SS group, two infant weighed more than 2500 gm (Table 9).

**Table 9:** Showing distribution of birth weight in study and control groups AA: Control group with normal Hb, AS: Sickle cell trait, SS: Sickle cell anemia/diseases

GROUP	<1000gm		1000-1500gm		1500-2000gm		2000-2500gm		>2500gm	
	No.	%	No.	%	No.	%	No.	%	No.	%
AA (n=100)	0	0	0	0	13	13	36	36	51	51
AS(n=187)	4	2.14	6	3.21	18	9.63	97	51.87	62	33.15
SS(n=38)	0	0	3	7.89	15	39.47	18	47.37	2	5.26

**Figure 1:**
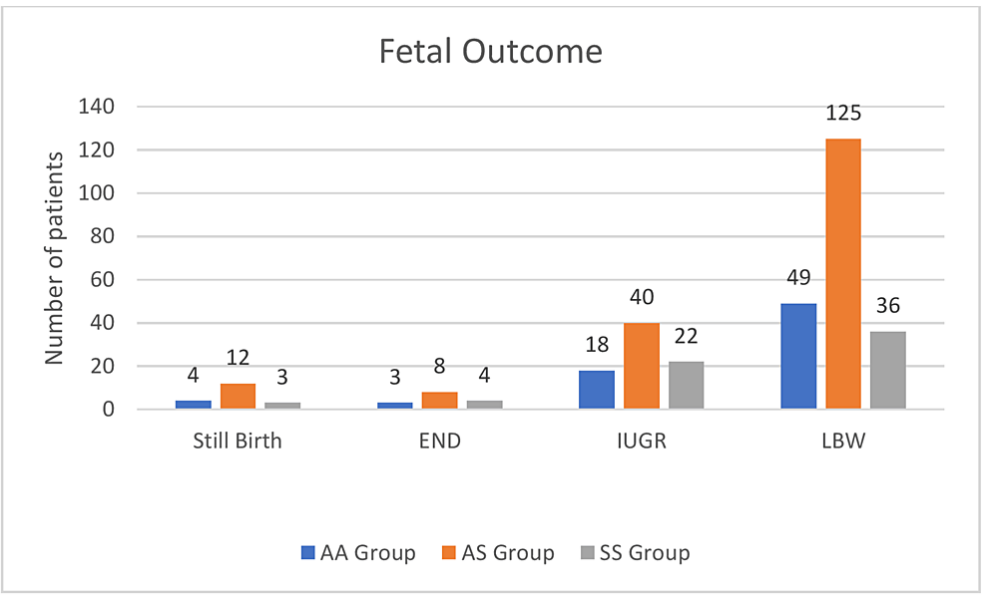
Graphical summary of fetal outcome in three groups AA: Control group with normal Hb, AS: Sickle cell trait, SS: Sickle cell anemia/diseases, END: Early neonatal death, IUGR: Intrauterine growth restriction, LBW: Low birth weight

Incidence of growth-retarded babies was significantly higher in the SS group at 57.89% as compared to 18% in the AA group and 21.39% in the AS group. There were three stillbirths in the SS group, but four early neonatal deaths were seen. Perinatal mortality rate was 16.13% in the SS group and 9.37% in the AS group compared to 6% in the control group. We did not record a significant difference between the groups with regard to stillbirth. However, the rates of IUGR were higher among women in the SS group (57.89%) compared with the normal Hb (AA) group at 18%, P = 0.001 (Table 10). Graphical representation of fetal outcome in all three groups has been shown in Figure 1.

**Table 10:** Fetal outcome in all groups AA: Control group with normal Hb, AS: Sickle cell trait, SS: Sickle cell anemia/diseases, END: Early neonatal death, IUGR: Intrauterine growth restriction, LBW: Low birth weight

GROUP	AA (n=100)			AS (n=187)			SS (n=38)		
	No.	%	P value	No.	%	P value	No.	%	P value
Stillbirth	4	4	-	12	6.42	-	3	7.89	0.672
END	3	3	-	8	4.28	-	4	10.53	0.036
IUGR	18	18	0.024	40	21.39	0.012	22	57.89	0.001
LBW	49	49	0.001	125	66.84	0.002	36	94.73	0.001

Logistic regression analysis confirmed that HbSS (AOR = 1.72, 95% CI = 1.18-2.49, P = 0.006) and HbAS women (AOR = 2.67, 95% CI = 1.52-4.13, P < 0.001) were more likely to undergo CS delivery compared with the comparison group (Table 11). Infants delivered by HbSS women were more likely to experience fetal distress (AOR = 4.13, 95% CI = 1.39-7.56, P = 0.003). More stillbirth was recorded in HbSS (AOR = 2.67, 95% CI = 1.19-4.99, P = 0.002) as compared to HbAS women (AOR = 2.29, 95% CI = 1.09-4.66, P = 0.036). Surprisingly, risk of delivering LBW babies was seen less in HbAS (AOR = 0.32, 95% CI = 0.05-0.71, P = 0.013) compared with HbSS and normal (Table 11).

**Table 11:** Logistic regression analysis of maternal and perinatal outcomes among women with sickle cell disease The adjusted odds ratio (AOR) and 95% confidence interval (CI) were taken with logistic regression in multivariate analyses adjusted for age, parity, and other variables in the study. Model I was used for the logistic regression analysis. The reference for cesarean section was vaginal delivery. LSCS: lower segment cesarean section, LBW: low birth weight; NICU: neonatal intensive care unit

Diagnosis	Hb SS			Hb AS		
	AOR	95% CI	P value	AOR	95% CI	P value
LSCS	1.72	1.18–2.49	0.006	2.67	1.52–4.13	< 0.001
LBW	0.75	0.41–1.73	0.6109	0.32	0.05–0.71	0.013
Premature	1.13	0.77–2.03	0.656	1.03	0.51–2.02	0.678
NICU admission	1.74	0.84–2.66	0.059	1.09	0.53–2.23	0.765
Labored Respiration	1.07	0.22–4.09	0.877	–	–	–
Fetal Distress	4.13	1.39–7.56	0.003	0.73	0.19–3.22	0.761
Asphyxia	1.05	0.68–4.23	0.777	1.82	0.44–6.33	0.563
Still born	2.67	1.19–4.99	0.002	2.29	1.09–4.66	0.036

There were four maternal deaths in the study group, two in the AS group and two in the SS group, as compared to no death in the control group. The maternal mortality rate in the SS group was statistically significant as compared to the control group (p value < 0.05). Thus, the maternal mortality rate in the present series was 5.26% in the SS group and 1.07% in the AS group (Table 12).

**Table 12:** Maternal mortality rate in present series AA: Control group with normal Hb, AS: Sickle cell trait, SS: Sickle cell anemia/diseases

Group	Total No. Of cases	Total No. Of deaths	Percentage	P value
AA group	100	0	0	
AS group	187	2	1.07	>0.05 Non significant
SS group	38	2	5.26	<0.05 Significant

Puerperium

One patient (1%) in the AA group, two patients (1.07%) in the AS group, and four patients in the SS group (10.52%) had puerperal sepsis. Four patients in the SS group (10.52%) patient had vasoocclusive crisis.

## Discussion

It is a well-known fact that pregnant women with SCD have a greater risk to develop maternal and fetal complications during pregnancy compared with healthy women [4-8]. In developing countries like those in Africa, a mortality rate of 7-12% persists [20,21] and in India it is 4-40% [22-24] as a result of SCD in pregnancy, because of poor and insufficient antenatal care especially in remote areas.

The results of our study were consistent with other published data. Similar to our results, Hickman et al. [25] and Barfield et al. [26] reported that they estimated around 1% of all pregnancies had SCD. Anaemia is highly prevalent among pregnant women in India and in addition, women with SCD poses greater maternal and fetal health problem [27-29].

The present study showed that women with homozygous SCD have a greater risk of pre-term birth, stillbirth, and low birth weight compared to the SCD trait and non-SCD pregnancies. Smith-Whitley [30] and Muganyizi and Kidanto [31] have reported similar findings; SCD deliveries have a higher chance of lower birth weights, low gestational period and increased stillbirth rate compared to normal deliveries. In women with sickle cell anemia, pregnancy possesses a greater chance to develop sickle cell crisis and increased rate of hospitalization. Many authors reported that sickle cell crisis took place in 50% of pregnant women with SCD and caused mortality [4,32-34]. However, in our study we recorded 44.74% of sickle cell crises, which is similar to another published study [35]. Multiple studies have reported a high risk of abortion among SCD women [36-38]. In our study, we excluded abortions.

Unlike studies in developed countries [12,26,39], the present study reported a significantly higher maternal mortality rate in women with SCD as compared with non-SCD women. The maternal mortality rate was 5.26% in the sickle cell anemia (SS) group which is similar to other developing countries that indicate poor antenatal, perinatal and post-natal care.

The reason for preterm delivery among women with SCD is still not clear, many published data have shown an increased risk of preterm delivery among such women [40,41]. However, in our study 21.05% of cases of preterm delivery were seen in the SS group. The CS deliveries were performed more in women with SCD than non-SCD women. In foreseen fetal compromise and previous obstetrical history, the deliveries by CS are preplanned in order to minimize further obstetrical complications during vaginal delivery which may occur. Constant fetal monitoring like fetal heart rate may also help surgeons to decide on CS [4]. The main indication to perform CS deliveries was fetal distress, failed labor progression or bad previous obstetrical history. Many authors recorded that women with SCD have a greater risk of IUGR [42-45].

In our study the prevalence of IUGR was higher in women with HbSS than HbAS and the control group. Moreover, HbSS women were at increased risk of IUGR as compared with normal women. This increased risk could be because of long-standing maternal anemia, hypercoagulation state during pregnancy, degree of sickling and vaso-occlusion which compromise placental circulation and lead to uterine growth restriction [46,47]. IUGR is also due to reduction of oxygen content in blood, especially in anemic women which in turn reduces or affects placental perfusion [48]. Yu et al. noted that HbSS women were at a greater risk of being anemic. Haemolysis or hypersplenism was responsible for high prevalence of anemia in women with sickle cell disease [49]. More complications such as premature rupture of membrane (PROM), preterm delivery, spontaneous preterm labour, and LBW infants are also recorded in women with anemia by many authors [50,51]. These complications are responsible for an increased rate of perinatal morbidity and mortality, and an increased infant mortality rate (IMR).

In the present study we recorded an increased risk of intrauterine fetal demise (IUFD) among HbSS women. Women with SCD had a greater risk of intrapartum stillbirth than the control group. The exact pathological reason for intrapartum stillbirth and IUGR in these women is still not clear [49]. A few studies indicate that vaso-occlusion in the placenta, placental infarction, and placental insufficiency cause reduced nutrient supply and poor metabolic exchange responsible for IUFD and IUGR [52,53]. Moreover, stillbirth and a high incidence of PIH are also reported in women with SCD due to chronic antenatal anemia [52]. In the present study we recorded UTI in 8% of the AA group, 10.16% of the AS group and 23.68% of the SS group. Similarly, Thame et al. [53] and Seoud et al. [54] recorded that women with SCD have a higher risk of developing UTIs and placenta previa.

We recorded 11% mild PIH in the control group (AA group) and in the AS and SS groups it was severe PIH at 11% and 16% respectively. They also noted a high incidence of PIH in women with HbSS as compared with control. In the present study, preeclampsia was seen in 11% of the AS group and 16% of the SS group. Many authors have recorded a higher prevalence of preeclampsia in women with HbSS than in normal control [54-58]. The risk of preeclampsia is higher in women with HbSS than in women with HbAS. Larrabee and Monga [55] noticed in their study that 24.7% of sickle-positive women developed preeclampsia, whereas Cudihy and Lee [59] recorded it was as high as ten times more among women with HbSS than the control group.

In order to minimize adverse maternal and fetal outcomes, identification of women with higher risk, regular third-trimester screening for foetal growth, women with SCD, and management of anemia is important [60,61]. In a recent meta-analysis, Oteng-Ntim et al. observed that there is a need to research effective interventions and management in every secondary and tertiary care hospital to improve maternal outcome and minimise complications among women with SCD in developing countries [62].

Limitations of the study

There are a few limitations of the present study. This is a retrospective study based on patients’ data collected at a hospital and analyzed. Our study did not cover women with SCD who delivered at home. Therefore, the finding of this study may not be generalized to those women who delivered at home. The study is based on pregnant women admissions only however the present study did not cover those pregnant women who were admitted with other problems not related to sickle cell.

## Conclusions

The present study reported a greater risk and adverse pregnancy outcomes in women with SCD as compared to sickle cell trait and normal women. Pregnancies with combined SCD and anaemia possess a greater challenge in achieving better maternal and fetal outcomes in developing countries. To minimize maternal and fetal complications in such women, multispecialty team management is required.

It is advisable to diagnose sickle cell anemia before conception or during early pregnancy. Maternal morbidity and mortality and perinatal mortality are high in spite of a pronounced decrease due to improving care. Periodic visits with the obstetrician and hematologist are mandatory. Risks are highest in late pregnancy, during delivery and in the postpartum period. However, the whole pregnancy period is high risk and requires close monitoring.

## Appendices

**Table 13:** Proforma used in study

PROFORMA												
PREGANCY AND SICKLE CELL DISEASE												
NAME:			AGE AND SEX:					ADDRESS:				
HEMOGLOBIN PATTERN (BY HPLC)												
SS/AS/AA												
GRAVIDA AND PARITY												
HEMOGLOBIN PATTERN OF HUSBAND (BY HPLC)												
OCC. OF HUSBAND												
NUTRITIONAL STATUS												
OBESE/ASTHENIC/WELL- NOURISHED												
OCCUPATION												
MATERNAL HEALTH												
ANTEPARTUM			MENSTRUAL HISTORY					AGE OF MENARCHE: <16yrs/ 16-18Yrs/>18Yrs				
								RHYTHM: <28 days / 28-31days: >31days				
								DURATION OF FLOW: <2days/ 4-6 days/ >6 days				
								TYPE OF FLOW: None/ Spotting/ Regular sanitary				
								ASSOCIATED SYMPTOMS: Bloating of abdomen Premenstrual pain/ Headache / Nausea/Body Tenderness/ Mood changes.				
								LMP				
								EDD				
			(B)GROWTH AND DEVELOPMENT					PRE- PREGNANCY WT.: Kg				
								PRESENT WT.: 1^ST ^TRIMESTER: 2^ND ^TRIMESTER: 3^RD ^ TRIMESTER:				
								SYMPTOMS: Nausea/ Appetite / Presence of oedema/ Meal frequency / Satiety/ Dyspepsia/Constipation / Foul smelling stool / Others				
			(C) OBSTETRIC HISTORY			PERSONAL HISTORY		Time since marriage				
								H/o Previous admission				
								Family history: Total Members H/o SCD in family				
								Contraception: Barrier: Condom / Spermicidal Jelly/ Diaphragm OCP: Combined pills / Low dose oestrogen IUD: CuT				
								Alcohol/Tobacco consumption				
					OBSTETRIC HISTORY							YR & DATE
S.NO.	NO. OF PREGNANCY	Year& Date	PLACE OF CONFINEMENT	DURATION OF GESTATION			PREGNANCY EVENTS 1.ABORTION 2.PREMATURE LABOUR		LABOR EVENTS *S/I/C/F C: Elective/ Emergency			
									1.												
2.												
3.												
4.												
5.												
6.												
*S-spontaneous/I-Induced/C-Caesarean Section/F-Forcep delivery												
BLOOD TRASFUSION REQ.:		no. of blood units transfused										
		prophylactic/exchange/ postpartum:										

	(D) PRESENTING COMPLAINTS	ANEMIA : Mild / Moderate / Severe	
		PAINFUL CRISIS	PRECIPITATING FACTORS: Cold/ Dehydration / Infection/ Menses/ Emotional outcome/ Strenuous work/ Diarrhoea/ High altitude/Alcohol consumption.
			BODY PAIN SITE: Humerus / Tibia/ Femur / Spine/ Rib cage / Elbow/ Knee/ Digits/ Redness/ Warmth Tenderness Symmetrical / Asymmetrical
			BODY PAIN TYPE: Throbbing / Excruciating Constant / Intermittent Affected by movement/ Posture Awake at night Interfere with daily activity Localized / diffuse
			RELIEVED BY:Medication at home/ OPD
			HOSPITATLIZATION: Yes/No No. of time / Year.:
		HEMORRHAGE <28Wks of pregnancy:	ABDOMINAL PAIN: Vague / Specific site
			SPECIFIC SITE: Quadrant: Upper Right/Left Lower Right/Left
			ASSOCIATED WITH: Nausea / Vomiting / Fever/ Persistence of bowel sounds / related with diet, exercise, sleep / Yellowness of sclera, urine /
		>28wks of pregnancy:	Placenta Praevia: Breech/ Transverse lie
			Premature labour :Spontaneous / induced
			Abruptio placenta: Revealed / Conceal
		. GENITOURINARY COMPLAINTS	UTI(urinary tract infection): Burning sensation in micturition/increased urge to void/ stress incontinence / fever with chills and rigor / vomiting/ diarrhoea/ increased awareness of heart beat / generalized muscle tenderness
			Associated symptoms: Recent sore throat/ Fever/ Chills and rigor/Urine Frequency / Urgency / Urine flow flank pain radiating to groin vaginal discharge/ Bowel habits / Any other
		PIH (TOXEMIA / PREECLAMPSIA)	<20 WKS of Pregnancy/>20 WKS of Pregnancy.:
			Prior history of similar episode: Yes/No
			Clinical features: B/P: < 140/90 / >140/90
			Swelling slight/ generalized
			Pre-pregnancy weight:
			Gain in weight:
			Proteinuria: Yes / No
			Associated with: Posture / Exposure/ Emotional stress /Cold / Fever/ CHF/ Other
		ECLAMPSIA	Feature of Preeclampsia: Yes/No
			Convulsion: Generalized tonic clonic: Yes /No Antepartum/Intrapartum/Postpartum
		ACUTE CHEST SYNDROME	C/f: Chest pain/ fever/ Difficulty in breathing / Cough / New pulmonary infiltrate of X-ray / other
			Hospitalization: Yes/ No.
			No of time:
2)INTRAPARTUM PERIOD	PRE-TERM LABOUR	<28Wks Of Pregnancy	: Yes/ No
		Precipitating factor: Prior history of abortion / Asymptomatic bacteriuria / UTI / Preeclampsia/ Multiple pregnancy / PROM(premature rupture of memberane)	
	CAESAREAN SECTION	Elective / Emergency	
3)POSTPARTUM PERIOD	ECLAMPSIA	Yes / No	
	PUERPERAL SEPSIS	Fever: Mild / Mod / Severe	
		Fever: <10days PPP / > 10 days PPP(post partum period)	
		Fever with chills and rigor	
		Lochia: Offensive / Odourless Scant / Copious Red/ Brown	
	PERINEAL WOUND	Repaired: Yes/No	
	PULMONARY SEPSIS	Yes/No	
	PULMONARY EMBOLISM	Yes/No	
	SICKLE CELL CRISIS	YES/NO	
FETAL OUTCOME			

IUGR(intra uterine growth retardation)	SGA: (small for gestational age)	YES / NO
		Pre-term / At – term
		2.5kg. birth / >2.5 kg birth
		previous pregnancy with child LBW < 2.5kg. Yes/ No If yes -------- how many with wt.
IUD(intrauterine death)	IUD	> 28 wks. Yes / no
		Previous Pregnancy with similar IUD Yes / No
		If yes ----- how many with wt.
LIVE BIRTHS	LIVE BIRTHS	How many births
		weight of each.
		Complication if any
		Transfusion if reqd.
